# Development and implementation of a quality improvement toolkit, iron deficiency in pregnancy with maternal iron optimization (IRON MOM): A before-and-after study

**DOI:** 10.1371/journal.pmed.1002867

**Published:** 2019-08-20

**Authors:** Jameel Abdulrehman, Andrea Lausman, Grace H. Tang, Rosane Nisenbaum, Jessica Petrucci, Katerina Pavenski, Lisa K. Hicks, Michelle Sholzberg

**Affiliations:** 1 Division of Hematology, Department of Medicine, University Health Network, Toronto, Canada; 2 Department of Obstetrics and Gynecology, St. Michael’s Hospital, University of Toronto, Canada; 3 Hematology Oncology Clinical Research Group, St. Michael’s Hospital, Toronto, Canada; 4 Li Ka Shing Knowledge Institute, St. Michael’s Hospital, Toronto, Canada; 5 Applied Health Research Centre, MAP Centre for Urban Health Solutions, St. Michael’s Hospital, University of Toronto, Canada; 6 Division of Hematology/Oncology, Department of Medicine and Department of Laboratory Medicine, St. Michael’s Hospital, Toronto, Canada; 7 Department of Laboratory Medicine and Pathobiology, St. Michael’s Hospital, University of Toronto, Canada; Cornell University, UNITED STATES

## Abstract

**Background:**

Iron deficiency (ID) in pregnancy is a common problem that can compromise both maternal and fetal health. Although daily iron supplementation is a simple and effective means of treating ID in pregnancy, ID and ID anemia (IDA) often go unrecognized and untreated due to lack of knowledge of their implications and competing clinical priorities.

**Methods and findings:**

In order to enhance screening and management of ID and IDA in pregnancy, we developed a novel quality-improvement toolkit: ID in pregnancy with maternal iron optimization (IRON MOM), implemented at St. Michael’s Hospital in Toronto, Canada. It included clinical pathways for diagnosis and management, educational resources for clinicians and patients, templated laboratory requisitions, and standardized oral iron prescriptions. To assess the impact of IRON MOM, we retrospectively extracted laboratory data of all women seen in both the obstetrics clinic and the inpatient delivery ward settings from the electronic patient record (EPR) to compare measures pre- and post-implementation of the toolkit: a process measure of the rates of ferritin testing, and outcome measures of the proportion of women with an antenatal (predelivery) hemoglobin value below 100 g/L (anemia), the proportion of women who received a red blood cell (RBC) transfusion during pregnancy, and the proportion of women who received an RBC transfusion immediately following delivery or in the 8-week postpartum period. The pre-intervention period was from January 2012 to December 2016, and the post-intervention period was from January 2017 to December 2017. From the EPR, 1,292 and 2,400 ferritin tests and 16,603 and 3,282 antenatal hemoglobin results were extracted pre- and post-intervention, respectively. One year after implementation of IRON MOM, we found a 10-fold increase in the rate of ferritin testing in the obstetric clinics at our hospital and a lower risk of antenatal hemoglobin values below 100 g/L (pre-intervention 13.5% [95% confidence interval (CI) 13.0%–14.11%]; post-intervention 10.6% [95% CI 9.6%–11.7%], *p* < 0.0001). In addition, a significantly lower proportion of women received an RBC transfusion during their pregnancy (1.2% pre-intervention versus 0.8% post-intervention, *p* = 0.0499) or immediately following delivery and in the 8 weeks following (2.3% pre-intervention versus 1.6% post-intervention, *p* = 0.0214). Limitations of this study include the use of aggregate data extracted from the EPR, and lack of a control group.

**Conclusions:**

The introduction of a standardized toolkit including diagnostic and management pathways as well as other aids increased ferritin testing and decreased the incidence of anemia among women presenting for delivery at our site. This strategy also resulted in reduced proportions of women receiving RBC transfusion during pregnancy and in the first 8 weeks postpartum. The IRON MOM toolkit is a low-tech strategy that could be easily scaled to other settings.

## Abstract 2

Michelle Sholzberg and colleagues report on the implementation of a toolkit aimed at improving management of anemia in pregnancy.

## Author summary

**Why was this study done?:**

- Iron deficiency is a common problem in pregnancy, with serious health consequences including maternal anemia requiring blood transfusions, early labor, low birth weight, and long-term developmental issues in the child.
- If detected early, iron deficiency in pregnancy can be treated with convenient and inexpensive daily oral iron pills.
- Unfortunately, due to low awareness and other competing medical issues during pregnancy, iron deficiency often goes undiagnosed and untreated.

**What did the researchers do and find?:**

- We created a toolkit including treatment algorithms, educational information for doctors and patients, modified blood work requisitions, and standardized iron prescriptions in order to help obstetricians detect and treat iron deficiency.
- We found that after implementation of the toolkit, there was an increase in testing for iron deficiency, a decrease in maternal anemia, and a decrease in maternal blood transfusions during and after pregnancy.

**What do these finding mean?:**

- The implementation of the toolkit successfully helped obstetricians detect and treat iron deficiency in pregnancy.
- The toolkit is a low-tech strategy that could be adapted to be used in other patient groups with iron deficiency.

## Introduction

Iron deficiency (ID) in pregnancy is a common problem that can have serious consequences. At baseline, 9% to 11% of teenage girls and women of child bearing age in developed countries have ID [1]. Individuals from lower income and of minority groups are more commonly affected [1]. The risk of ID increases during pregnancy due to an increase in maternal iron requirements to accommodate the expansion of maternal red blood cell (RBC) mass, development of the placenta and fetus, and the loss of blood associated with labor and delivery [2]. Unfortunately, women cannot meet these iron demands through food intake alone and must rely upon iron stores [3]. If the stores are suboptimal, secondary anemia ensues [4]. ID is the most common cause of anemia in pregnancy [3,4]. ID anemia (IDA) is associated with preterm delivery, cesarean delivery, RBC transfusions, low birth weight, 5-minute Apgar score <7, neonatal intensive care unit admission, and long-term effects on mental and psychomotor development in the child [2,5–8].

Fortunately, early detection of ID and treatment with daily iron supplementation is a straightforward and effective method of addressing ID. ID and IDA can be detected using simple blood tests of hemoglobin and ferritin [9]. Guidelines from the World Health Organization (WHO), the Centers for Disease Control and Prevention, and the American College of Obstetricians and Gynecologists (ACOG) all recommend screening for anemia in pregnancy [10–12]. However, the lack of anemia does not imply adequate iron stores, as up to 59% of women with ID are not anemic [13].

Oral iron supplementation is inexpensive, convenient, available in various preparations, and, most importantly, effective [9]. In a meta-analysis, daily oral iron supplementation in pregnant women decreased the risk of term maternal anemia (risk ratio [RR] 0.30, 95% confidence interval [CI] 0.19–0.46, 14 trials, 2,199 women), term maternal IDA (RR 0.33, 95% CI 0.16–0.69, 6 trials, 1,088 women), and term ID (RR 0.43, 95% CI 0.27–0.66, 7 trials, 1,256 women) [14].

Although ID in pregnancy is simple to diagnose and treat, it often goes undiagnosed and untreated, due to low awareness of its implications and multiple competing clinical priorities in busy obstetrical clinics. An Australian group recently published a multipronged quality improvement (QI) intitative focusing on optimizing management of ID in pregnancy and improving the management of postpartum anemia [15]. They found an increase in ferritin requests, a decrease in the rate of admission intrapartum anemia, and a decrease in postpartum RBC transufusions [15].

Our institution, St. Michael’s Hospital, is an inner city tertiary care center in Toronto, Canada, that delivers approximately 3,000 babies annually. We describe the development and implementation of a novel QI toolkit: ID in pregnancy with maternal iron optimization (IRON MOM), designed to improve early detection and management of maternal ID and IDA. The toolkit included clinical pathways, educational resources for clinicians and patients, adjusted laboratory requisitions, and standardized iron prescriptions. The toolkit was implemented to determine if priority setting and simple process changes could increase the rate of ferritin testing, decrease the proportion of women with an antenatal (predelivery) hemoglobin value below 100 g/L (anemia), and decrease RBC transfusion rates during and after pregnancy.

## Methods

### Setting

Our institution, St. Michael’s Hospital, largely serves a demographic from lower socioeconomic backgrounds [16]. Approximately 40% of the patients served by our institution are born in a foreign country, and approximately 20% are considered to be below low-income [16]. The obstetrical team at our institution includes 15 obstetricians at three outpatient clinics outside the hospital, but all deliveries occur at the hospital itself. The IRON MOM toolkit was implemented at one of the clinics in which 12 of the obstetricians provide care.

The reporting of this study is in keeping with the SQUIRE 2.0 guidelines [17].

### Intervention

As part of the multimodal IRON MOM QI initiative, we developed three distinct clinical pathways at each routine obstetrical clinical visit, developed educational resources, adjusted laboratory requisitions, and created standardized iron prescriptions. The toolkit was developed through multidisciplinary collaboration between Hematology, Obstetrics, Transfusion Medicine, Nursing, and Laboratory Medicine. The goal of the IRON MOM toolkit was to enhance screening and management of ID/IDA in pregnancy.

The overall initiative is described visually in S1 Fig, depicting the recommended actions and their timing. Each routine visit, at week 16, week 28, and postpartum, has a clinical pathway, guiding the clinician through the decision tree, using laboratory values of hemoglobin and ferritin to ascertain if initiation of oral iron supplementation is appropriate and at what dose (see S2 Fig, S3 Fig and S4 Fig).

Educational materials were developed for obstetrical teams and patients (see S5 Fig, S6 Fig and S7 Fig). For the obstetrical teams, a list of oral iron supplements available in Ontario, including generic and trade names, as well as the relative cost per tablet or capsule, was devised. As the recommended doses of daily oral iron for treatment of ID in pregnancy varies from 60 to 200 mg of elemental iron, a variety of options for oral iron supplementation were listed in the educational materials and clinical pathways [10,18]. Posters of the clinical pathways were displayed in communal physician work spaces in the obstetric clinic. For patients, two separate patient educational handouts were created. The first was distributed to all patients prior to testing for ID, which described the role of iron in pregnancy, and how ID is tested for. The second handout was distributed only to those who were found to have ID, and described its treatment. Both patient educational handouts were developed in conjunction with the Patient and Family Education Program at our institution to ensure that the language used was accessible to the patient population we serve. Due to budgetary constraints, materials were only made available in English.

The laboratory requisitions used by the obstetrics team were modified to encourage ferritin testing, as the check box for ferritin was moved to a more prominent area at the top of the sheet, near the complete blood count (CBC) check box. However, ferritin was not automatically checked.

Standardized iron prescription pads with all the various iron supplementations used in the initiative were provided to the obstetrics team. Although oral iron supplementation is available over the counter, having a prescription would enforce the importance of iron supplementation, serve as a reminder to the patient, and, for some women, would allow for their insurance plan to cover the cost of the drug.

Plan-Do-Study-Act (PDSA) cycles were conducted to improve the IRON MOM at each iteration [19].

The total costs of developing and implementing the IRON MOM toolkit were low. All the materials were developed by our multidisciplinary team. The total cost of printing the materials for the toolkit was approximately CAN$500 to CAN$1,000. With the implementation of the toolkit, we predicted a large increase in ferritin testing. The average cost at our institution for a ferritin test, including labor and supplies, was CAN$0.72 per test. The toolkit was streamlined to minimize the time spent on the initiative by the obstetrician per patient.

### Study design

To measure the influence of the toolkit at our institution, we compared pre- and post-intervention aggregate laboratory data extracted from the electronic patient record (EPR), using a time series design [20,21]. The process and outcome measures were determined prior to development of the toolkit, but the formal analysis plan was devised after the toolkit had been implemented for one year and is detailed below. The pre-intervention period encompassing January 1, 2012, to December 31, 2016, was compared with the post-intervention period, January 1, 2017, to December 31, 2017.

### Process and outcomes measures

We decided on one process measure and three outcome measures to compare pre- and post-intervention. Our process measure was the rate of ferritin testing in the obstetric clinics. We selected ferritin testing as our process measure, as a low ferritin is the best test for ID in pregnancy, with higher sensitivity and specificity than transferrin saturation, serum iron, or erythrocyte protoporphyrin values [3]. Our outcome measures were the proportion of women with an antenatal hemoglobin value below 100 g/L, the proportion of women who received an RBC transfusion during pregnancy, and the proportion of women who received an RBC transfusion immediately following delivery or in the 8-week postpartum period. We chose an antenatal hemoglobin value below 100 g/L as an outcome measure as a midway point between WHO and ACOG definitions of anemia in pregnancy (hemoglobin <110 g/L in the first and third trimester, and <105 g/L in the second trimester) and the lower hemoglobin values of 70 to 80 g/L, in which physicians would likely consider RBC transfusion [11,22]. We chose RBC transfusions during pregnancy, as well as immediately following delivery or in the 8-week postpartum period, as outcome measures to account for iron depletion due to both increases in maternal iron requirements and the loss of blood associated with labor and delivery [2].

### Statistical analysis

From January 1, 2012, to December 31, 2017, laboratory data of all women seen in both the obstetrics clinic and the inpatient delivery ward settings were extracted from the EPR. Data extracted include ferritin and hemoglobin (from the CBC) values. All ferritin requests at the obstetrics clinic were assumed to be ordered for purposes of ID screening. All CBC requests at the obstetrics clinic were assumed to represent a routine clinic visit. We considered the first hemoglobin measured during their hospitalization for delivery to be the antenatal hemoglobin. Women with multiple hospital admissions within a 280-day period had only the first hemoglobin measurement of their last admission for delivery included as their antenatal hemoglobin. Women with multiple pregnancies in the time periods of interest were included and were identified as having a new pregnancy by the timing of the delivery ward hemoglobin measurements being greater than 280 days apart. RBC transfusion data were obtained from the transfusion medicine laboratory and linked to women involved in the study through their medical record number.

For each study month, the rate of ferritin testing in the obstetric clinic was calculated as the number of total clinic ferritin tests divided by the total number of CBC tests, multiplied by 100, to simplify interpretation. Changes in monthly ferritin rates from pre- to post-intervention were evaluated using interrupted time series analysis [23–25].

Pre- and post-intervention distributions of antenatal hemoglobin values for all tests were initially compared using the chi-squared test when dichotomized. Considering each woman and each study period, antenatal hemoglobin values were recoded as 1 if any test value was <100 g/L or 0 if all tests yielded values ≥100 g/L. However, because women could be pregnant and provide data during both periods, we used generalized estimating equations with the binomial distribution and logit link to estimate the risk (predicted probability) of ever having values <100 g/L for pre- and post-intervention periods.

Considering the first pregnancy during the study period, the proportion of women who received at least one RBC transfusion pre-intervention compared with post-intervention was analyzed for two time periods, during pregnancy (within 10 months of delivery), and immediately following delivery or in the 8-week postpartum period, using the chi-squared test. Data were analyzed using SAS software version 9.4 (SAS institute, Cary, NC) and Stata 13.0 (StataCorp. 2013. *Stata Statistical Software*: *Release 13*. College Station, TX: StataCorp LP).

### Ethics

The IRON MOM initiative was formally reviewed by institutional authorities at St. Michael’s Hospital and deemed to neither require research ethics board approval nor written informed consent from participants. This was a local quality improvement project (QIP), and as such, the Privacy Office at St. Michael’s Hospital assumes the responsibility of reviewing projects and approving the use of Personal Health Information for the specific objectives outlined in our protocol. The privacy office prohibits access to data for this project, to protect patient privacy.

## Results

### Data collected

In total, there were 3,692 ferritin tests and 18,682 CBC tests in the obstetric clinic, and 19,885 antenatal hemoglobin tests available from the delivery ward during the entire study period. In the pre-intervention period from January 1, 2012, to December 31, 2016, there were 1,292 ferritin tests and 15,714 CBC tests from the obstetric clinic, and 16,603 antenatal hemoglobin tests from the delivery ward. In the post-intervention period from January 1, 2017, to December 31, 2017, there were 2,400 ferritin tests and 2,968 CBC tests from the obstetric clinic, and 3,282 antenatal hemoglobin tests from the delivery ward.

### Clinic ferritin requests

The pre-intervention period was composed of 60 months in which the mean number of ferritin tests in the obstetric clinic per month was 21.5 (standard deviation [SD] 10.6), the mean number of CBC tests in the obstetric clinic per month was 261.9 (SD 30.5), and the mean monthly rate of ferritin testing was 8.4 per 100 tests (SD 4.4 per 100).

The post-intervention period was composed of 12 months, in which the mean number of ferritin tests in the obstetric clinic per month was 200.0 (SD 25.3), the mean number of CBC tests in the obstetric clinic per month was 247.3 (SD 30.3), and the mean monthly rate of ferritin testing was 81.0 per 100 (SD 6.3 per 100).

**Fig 1:**
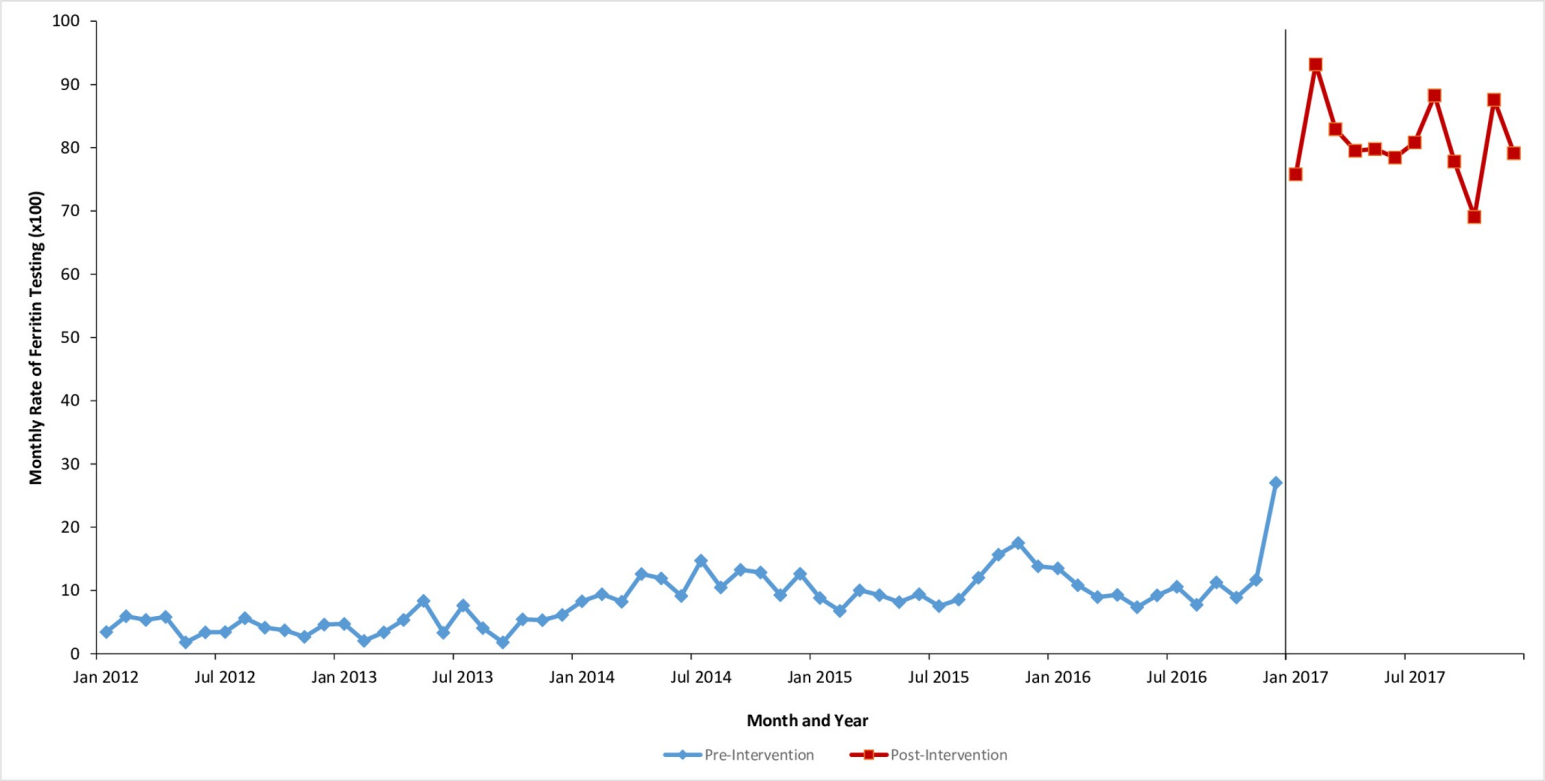
Monthly rate of ferritin testing.

Therefore, overall, the mean monthly rate of ferritin testing increased by almost 10-fold in the post-intervention period. Interrupted time series analysis indicated that in the first month of the intervention, there was an increase in mean ferritin rate of 69.0 per 100 (95% CI 62.2–75.9 per 100), which was statistically significant (*p* < 0.0001). Although post-intervention rates decreased over time, there was not a significant change, as expressed by the slope estimate of −0.3 per 100 (95% CI −1.2 to 0.6 per 100, *p* = 0.475) (see Fig 1).

### Antenatal hemoglobin

A total of 17,636 unique women were represented in the 19,885 delivery ward antenatal hemoglobin tests. Most women (*n* = 14,356 or 81.4%) were captured only in the pre-intervention period, 2,617 (14.8%) had data during the post-intervention period, while 3.8% (or 663) had data for both pre- and post-intervention periods. Using generalized estimating equations, we estimated that the risk of antenatal hemoglobin values less than 100 g/L was lower in the post-intervention period than in the pre-intervention period (10.6%, 95% CI 9.6%–11.7% and 13.5%, 95% CI 13.0%–14.1%, respectively, *p* < 0.0001).

### Proportion of women receiving at least one RBC transfusion

For this analysis, we only considered the first delivery during the study period; as such, 15,019 women were only counted once in the pre-intervention period and 2,617 women were counted in the post-intervention period. During pregnancy, 181 women out of 15,019 (1.2%) had at least one RBC transfusion pre-intervention compared with 20 out of 2,617 (0.8%) post-intervention (*p* = 0.0499). Immediately following delivery up to the 8-week postpartum period, 342 women out of 15,019 (2.3%) had at least one RBC transfusion pre-intervention compared with 41 out of 2,617 (1.6%) post-intervention (*p* = 0.0214).

## Discussion

One year after implementation of the paper-based IRON MOM toolkit, we found a large increase in the frequency of ferritin testing as an almost 10-fold increase in the mean monthly rate of ferritin testing in the obstetric clinics at our institution. There was a lower risk of an antenatal hemoglobin below 100 g/L and a lower proportion of women receiving an RBC transfusion during pregnancy and postpartum.

It is possible that the sudden substantial increase in the monthly rate of ferritin requests post-intervention represented a lack of knowledge among obstetricians on the prevalence of ID. Alternatively, the IRON MOM toolkit may have re-prioritized ID among the multiple competing management issues of pregnancy. Even in the month prior to the official launch, when the obstetrics team became aware of the upcoming initiative, the ferritin testing rate already started to increase. We believe this represented the initial culture change. This initiative demonstrated that priority setting by key champions and simple process changes in patient management can have enormous impact on key clinical outcomes.

Given that human gestation is 40 weeks, and our data analysis was limited to 52 weeks post-intervention, only a minority of women would have been exposed to the intervention through their entire pregnancy. Given the increased rate of ferritin testing, it seems likely that a greater improvement in antenatal hemoglobin values and a larger decrease in RBC transfusions will be seen going forward.

Clinically and statistically significant decreases in RBC transfusions were seen post-intervention in both the pregnancy and the 8-week postpartum time spans. However, it is possible that confounding variables not evaluated for in this study could have also changed during these time periods. An increased awareness of patient blood management, increased use of tranexamic acid at delivery, and/or improved surgical techniques and technologies could have contributed to this finding. However, we believe that IRON MOM was a key contributing factor. Furthermore, to simplify the statistical analysis, the 663 women who had deliveries in both the pre- and post-intervention periods were included only once in the pre-intervention period in the RBC transfusion assessment. Therefore, if they required RBC transfusions in both pre- and post-intervention periods, only the transfusion in the pre-intervention period would have been counted, and if they required an RBC transfusion only in the post-intervention period, the RBC transfusion would not have been included in the analysis. The end result is a bias towards fewer RBC transfusions recorded post-intervention. This is especially relevant, as women with rapid succession pregnancies, who likely had deliveries in both time periods, are at greater risk for IDA and RBC transfusions [26].

Compared with the Australian QI initiative, which focused on both ID in pregnancy and management of postpartum anemia, our initiative focused solely on ID in pregnancy [15]. Both initiatives included clinical pathways focusing on early identification and management of ID through assessments of ferritin and hemoglobin, educational resources for patients and physicians, and prescriptions for oral iron [15]. Both studies found an increase in ferritin testing and a decrease in maternal anemia using antenatal hemoglobin as the primary outcome measure [15]. Although the Australian study audited only a small group (434 women pre-intervention versus 55 women post-intervention), our study analyzed all antenatal hemoglobin values in the delivery ward available through our laboratory information system (16,603 pre-intervention versus 3,282 post-intervention) [15]. Lastly, both studies found a decrease in RBC transfusions post-intervention [15]. Therefore, our findings further emphasize that small changes in obstetrical patient management can substantially improve treatment of ID/IDA and maternal hemoglobin, and can decrease RBC transfusions, thereby decreasing complications of transfusions such as RBC alloimmunization and potential hemolytic disease of the fetus and newborn in future pregnancies [27].

There were several barriers that may have impacted the effect size of our QI initiative. First, although there are numerous benefits associated with oral iron supplementation, there are also notable side effects. Oral iron supplements are notorious for their gastrointestinal side effects, commonly nausea, vomiting, diarrhea, and constipation [9]. As these symptoms already predominate pregnancy, any medications that may worsen them may lead to treatment cessation and minimization of benefit. To address this, our educational tools provided tips on how to manage and minimize gastrointestinal symptoms. Furthermore, as the demographic at our institution is largely comprised of those of lower socioeconomic background, the extra financial burden from these iron pills may also pose a barrier. We included the relative costs of the particular iron supplements in our list of iron types and clinical pathways so that clinicians can advise their patients which iron formulation may best fit their economic disposition. Second, the initiative was completely paper based. Providing digital documents for either computer or mobile devices may have increased uptake and dissemination of the initiative among the obstetrical teams, improved buy-in, and reinforced the importance of treating ID among the pregnant women involved. Third, for this intervention to be successful, we relied heavily on the participation of the obstetrical team. Antenatal clinics are notoriously busy, with a fast pace, high workload, and numerous competing priorities. The IRON MOM initiative, although supported by the obstetrical team, may have been inadvertently omitted at a clinic visit if a higher priority item was identified. Despite these barriers, our findings were overwhelmingly positive. To minimize the effect of workload on the success of the intervention, we have focused on simplifying the process as much as possible through the availability of premodified blood work requisitions, directive clinical pathways, standardized iron prescriptions, standardized referral sheets, and patient materials that counsel on the appropriate administration of oral iron supplements. In addition, we sought feedback from the obstetrical team at the end of each PDSA cycle and made revisions in response. While the toolkit was generally well received by the obstetrics team, we did receive feedback to simplify the clinical pathways. Lastly, due to budgetary constraints, our educational materials were only made available in English. Toronto is a multicultural city with many spoken languages; thus, our educational material only being available in the English language may have limited its impact. However, the impact was likely minor, as only 4% of the patient demographic at our institution prefer healthcare information in a language other than English [16].

There were limitations to our study design. First, we lacked patient-level data. We extracted aggregate laboratory data from the EPR and made assumptions about why a blood test was being ordered, and at what time period in the pregnancy that test was done. There may have been hemoglobin or ferritin tests that did not meet our assumptions, but given the very large sample size, they are unlikely to have skewed the findings substantially. Second, our outcomes of clinic ferritin and antepartum hemoglobin were surrogate/laboratory markers; however, we believe that they associate strongly with important patient outcomes. Although ferritin can be elevated in the context of inflammation, thereby possibly obscuring a diagnosis of ID, the ID blood work was streamlined to only include hemoglobin and ferritin in order to simplify the algorithm for the obstetricians to increase utilization and uptake. Third, while the majority of obstetricians were involved in IRON MOM (12 of the 15 obstetricians at our institution), a minority were not, which likely attenuated the effect of the IRON MOM toolkit. Fourth, our study did not include an assessment of compliance with oral iron supplementation. Poor compliance with oral iron supplementation could also have attenuated the effect of the toolkit. Fifth, as this was a QI initiative, there was no comparator group nor randomization. Therefore, we could not control nor limit opportunities for bias.

In conclusion, the IRON MOM toolkit is an innovative means to address early detection and management of ID in pregnancy. The toolkit incorporates clinical pathways at each routine obstetrical clinical visit, educational resources, adjusted laboratory requisitions, and standardized iron prescriptions. IRON MOM simplifies and streamlines ID detection and management for clinicians and enhances patient education. The toolkit is simple to implement, with a potential for high impact, and is generalizable. It could be adopted for use in other obstetric care centers or adapted to serve other populations with a high burden of ID, such as nonpregnant gynecology patients or those with gastrointestinal malignancy. Future directions for IRON MOM include the implementation of a simplified digital format that is accessible on mobile devices, and the development of a large cluster-based randomized controlled trial to definitively assess the effect of IRON MOM.

## Supporting information

S1 Fig Iron optimization tasks. (PDF)

S2 Fig Week 16 clinical pathway. (PDF)

S3 Fig Week 28 clinical pathway. (PDF)

S4 Fig Postpartum clinical pathway. (PDF)

S5 Fig Patient educational material: Pretest. (PDF)

S6 Fig Patient educational material: Posttest. (PDF)

S7 Fig Physician educational material. (PDF)

## Abbreviations

ACOG: American College of Obstetricians and Gynecologists
CBC: complete blood count
CI: confidence interval
EPR: electronic patient record
ID: iron deficiency
IDA: ID anemia
IRON MOM: ID in pregnancy with maternal iron optimization
PDSA: Plan-Do-Study-Act
QI: quality improvement
QIP: quality improvement project
RBC: red blood cell
RR: risk ratio
SD: standard deviation
WHO: World Health Organization
