# Supplementary material for: Development and implementation of a quality improvement toolkit, iron deficiency in pregnancy with maternal iron optimization (IRON MOM): A before-and-after study
Source: PLoS Med. 2019 Aug 20;16(8):e1002867. doi: 10.1371/journal.pmed.1002867 (PMC6701755; doi:10.1371/journal.pmed.1002867)
Supplement: S1 Fig — (PDF) [file pmed.1002867.s001.pdf]

Iron Optimization Tasks

WEEK 12

Patient receives "Testing for Iron Deficiency in Pregnancy" patient education handout in pre-natal package

Order CBC and ferritin on blood work requisition

WEEK 16*

Review previous CBC and ferrritin

Consult week 16 clinical pathway

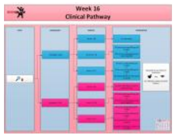

Give patient pre-printed iron prescription

And

"Iron Deficiency in Pregnancy – What You Need To Know" Patient Education Handout

WEEK 24-28

When testing for gestational diabetes, order CBC and ferritin on blood work requisition

WEEK 28*

Review previous CBC and ferrritin

Consult week 28 clinical pathway

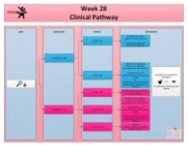

Give patient pre-printed iron prescription

And

"Iron Deficiency in Pregnancy – What You Need To Know" Patient Education Handout

L & D

Order CBC and ferritin

POST PARTUM

Review L&D ferrritin

Consult post partum clinical pathway

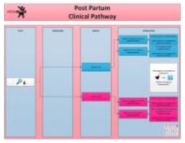

Give patient "Compliance Questionnaire"

* +/- 2 week
