# Supplementary figures and images for: Development and implementation of a quality improvement toolkit, iron deficiency in pregnancy with maternal iron optimization (IRON MOM): A before-and-after study
Source: PLoS Med. 2019 Aug 20;16(8):e1002867. doi: 10.1371/journal.pmed.1002867 (PMC6701755; doi:10.1371/journal.pmed.1002867)

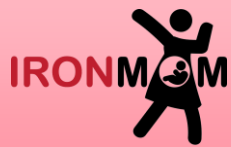

# Week 16 Clinical Pathway

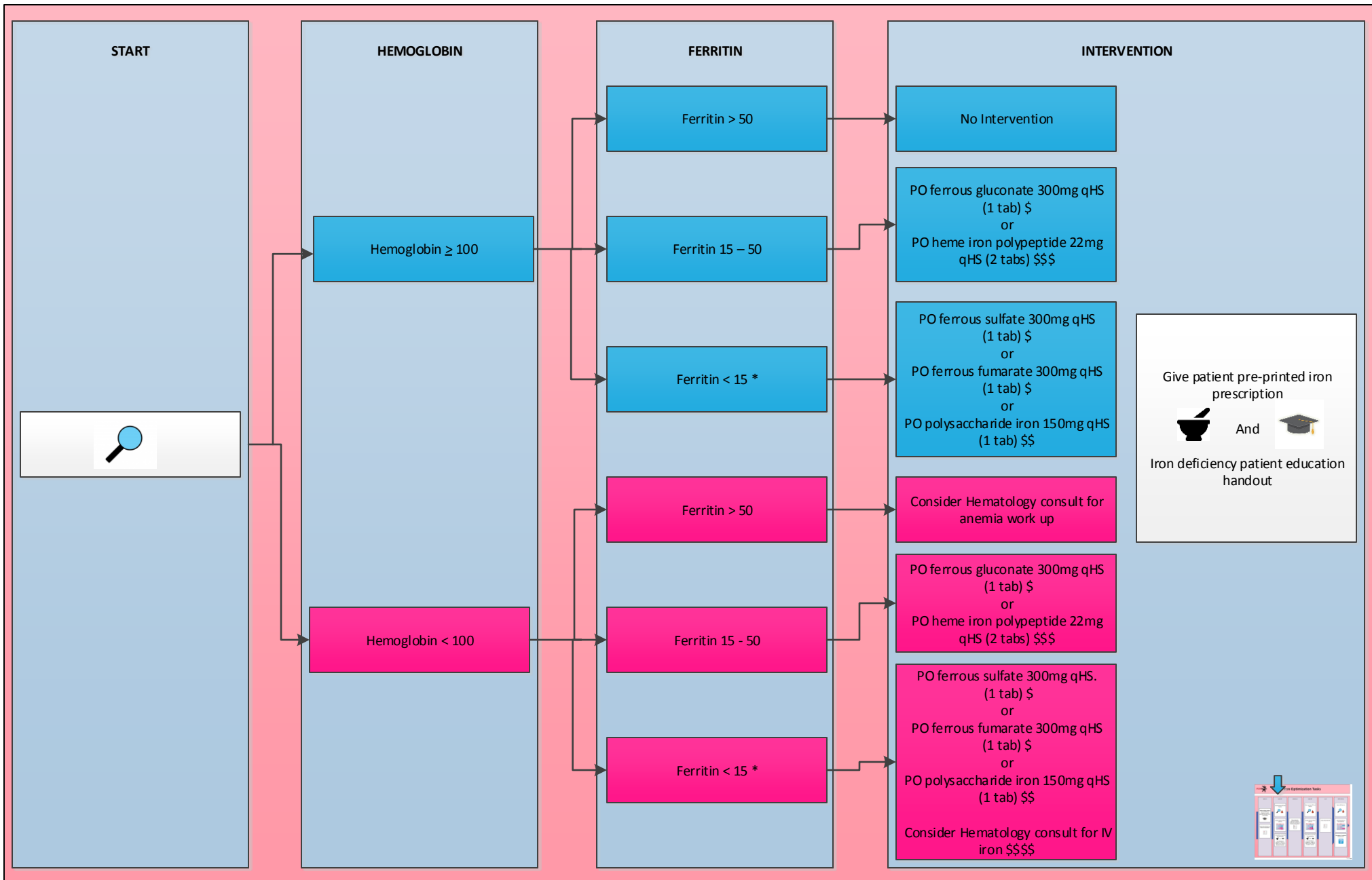

Supplement: S2 Fig — (PDF) [file pmed.1002867.s002.pdf]

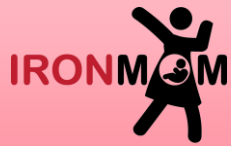

# Week 28 Clinical Pathway

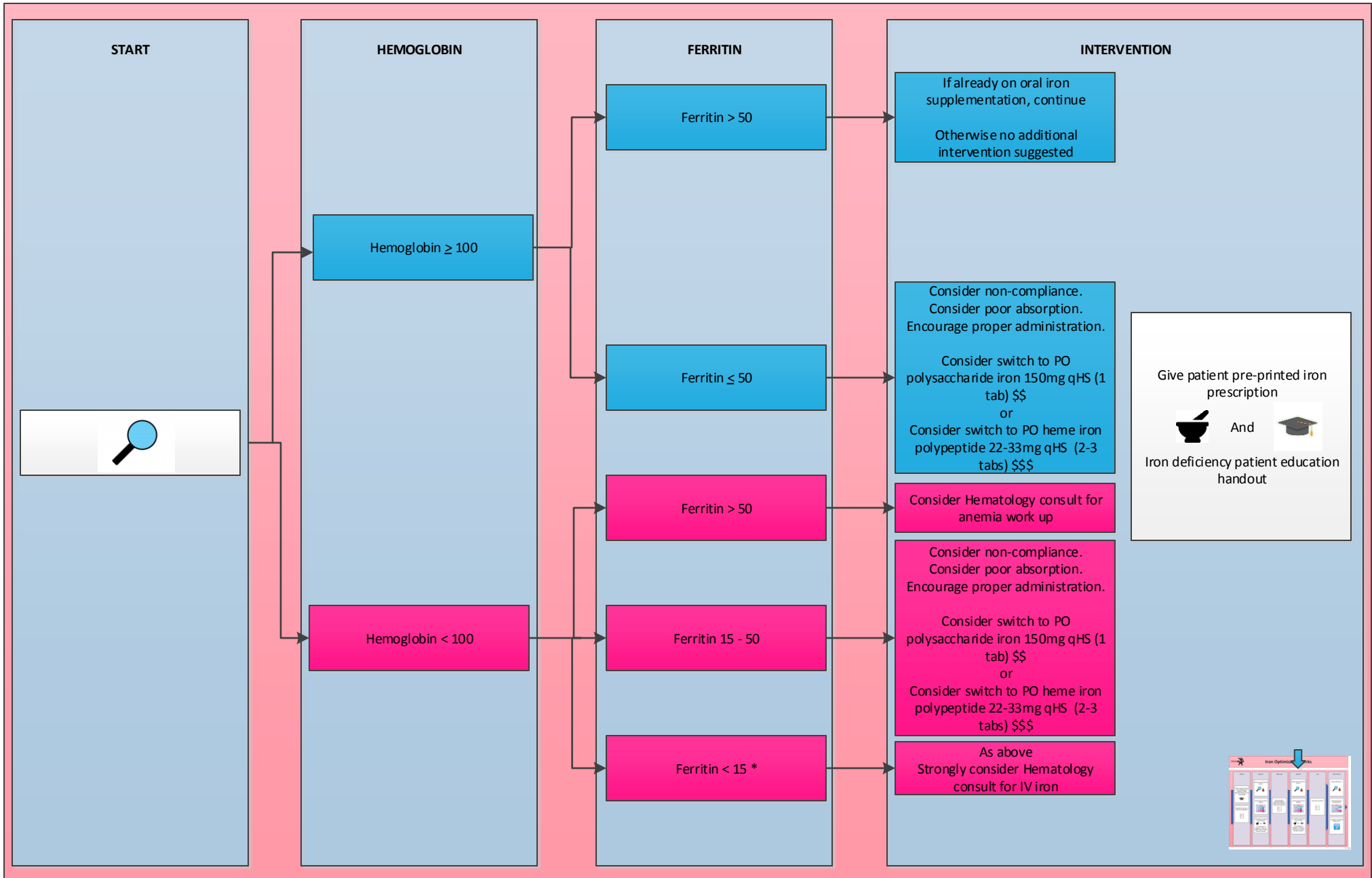

Supplement: S3 Fig — (PDF) [file pmed.1002867.s003.pdf]

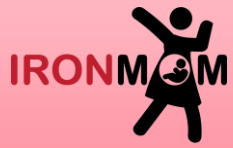

# Post Partum Clinical Pathway

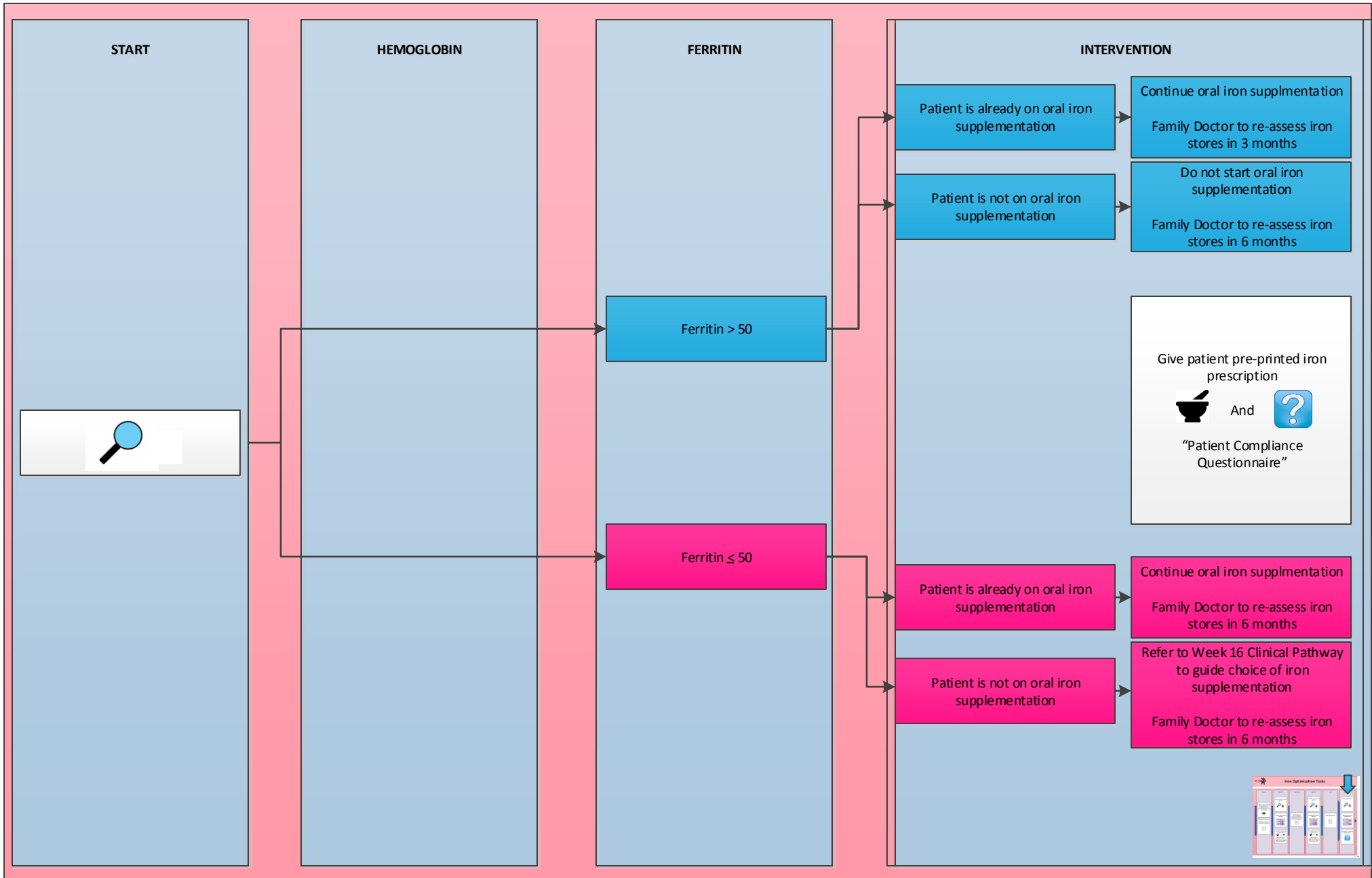

Supplement: S4 Fig — (PDF) [file pmed.1002867.s004.pdf]
