# Supplementary material for: Development and implementation of a quality improvement toolkit, iron deficiency in pregnancy with maternal iron optimization (IRON MOM): A before-and-after study
Source: PLoS Med. 2019 Aug 20;16(8):e1002867. doi: 10.1371/journal.pmed.1002867 (PMC6701755; doi:10.1371/journal.pmed.1002867)
Supplement: S5 Fig — (PDF) [file pmed.1002867.s005.pdf]

Testing for Iron Deficiency in Pregnancy

Testing for Iron Deficiency in Pregnancy

When you are 12 weeks pregnant, you will get a test for iron deficiency. Iron deficiency means having low levels of iron in your blood.

Iron is very important for you and your baby. For example, iron allows your blood to carry oxygen to your body and to your baby. Read this handout for more information.

What is iron deficiency?

Iron is a mineral that your body absorbs from the food you eat. When your body does not get enough iron (such as a diet low in iron or poor absorption in the gut) or loses too much (such as through menstrual periods), it becomes deficient in iron. If this happens over time, it can lead to anemia – low blood count.

Why is iron important during pregnancy?

When you are pregnant, your body has more demand for iron. Iron helps the baby and the placenta to develop. Your body needs iron to increase red blood cells and carry oxygen to the baby.

It is not always possible to get enough iron from food when you are pregnant. Many pregnant women need to take iron supplements.

If your store of iron continues to drop, you may develop anemia.

What are the risks of having anemia?

If you have anemia, this has risks for you and your baby.

You might :	Your baby might :
- Go into early labour<br>- Need a blood transfusion<br>- Have depression after delivery	- Weight less when born<br>- Develop more slowly

How common is this problem?

Iron deficiency is common. In western countries 4 out of every 10 women of child bearing age have iron deficiency.

For iron deficiency *anemia*, the rate is 4 out of every 100 women of child bearing age in western countries.

How will I know if I have iron deficiency?

We will do a blood test when you are 12 weeks pregnant. The test will check your levels of the following:

- Hemoglobin : The iron containing oxygen transporting protein in your red blood cells
- Ferritin : A protein that stores iron for use when needed

The test will show if you have iron deficiency. It will also show how low the levels are. Based on the results, your doctor will discuss your options for treatment.

For more information

If you have any questions or concerns, please talk to your doctor or primary health care provider.
