# Supplementary material for: Development and implementation of a quality improvement toolkit, iron deficiency in pregnancy with maternal iron optimization (IRON MOM): A before-and-after study
Source: PLoS Med. 2019 Aug 20;16(8):e1002867. doi: 10.1371/journal.pmed.1002867 (PMC6701755; doi:10.1371/journal.pmed.1002867)
Supplement: S6 Fig — (PDF) [file pmed.1002867.s006.pdf]

IRONMOM

# Iron Deficiency in Pregnancy
# What You Need to Know

## Iron Deficiency in Pregnancy– What you need to know

The results of your tests show that you have low levels of iron in your blood. This is called iron deficiency. If the levels are very low, this can lead to anemia - low blood count.

Iron is very important for the development of your baby. Read this handout to learn about:

- What is iron deficiency?
- How can iron deficiency affect me and my baby?
- How is it treated?

## What is iron deficiency?

Iron is a mineral that your body absorbs from the food you eat. When your body does not get enough iron (such as a diet low in iron or poor absorption in the gut) or loses too much (such as through menstrual periods), it becomes deficient in iron. If this happens over time, it can lead to anemia.

It is quite common for pregnant women to develop iron deficiency. When you are pregnant, your body has more demand for iron. Your body needs iron to increase red blood cells and carry oxygen to the baby. It is not always possible to get enough iron from food when you are pregnant.

## How can iron deficiency affect me and my baby?

Low levels of iron can present in many ways

| | |
|---|---|
| - Feeling tired<br>- Pale skin<br>- Nails that break easily or that have ridges<br>- Feeling dizzy<br>- Hair loss<br>- Weakness | - Twitches<br>- Feeling irritable or in a bad mood<br>- Restless leg syndrome - frequent urge to move your leg<br>- Pica - uncontrollable urge to eat things that are not food, such as dirt, soap, ice |

If you have anemia, this has risks for you and your baby.

| | |
|---|---|
| **You might :**<br>- Go into early labour<br>- Need a blood transfusion<br>- Have depression after delivery | **Your baby might :**<br>- Weight less when born<br>- Develop more slowly |

## How is iron deficiency treated?

It is very important to get treatment for iron deficiency. You and your doctor will talk about different ways to treat it. The treatment your doctor recommends will depend on the results of your tests.

There are 3 main ways to treat iron deficiency:

1. Eating more foods rich in iron
2. Taking iron supplements
3. Adding iron to your blood through IV tubes

### 1. Eating more foods rich in iron

There are two types of iron found in foods, heme and non-heme. While it's easier for your body to absorb heme sources of iron, it is important to eat foods with both types of iron.

Eat foods from both the lists below.

| Some foods with heme iron: | Some foods with non-heme iron: |
|---|---|
| - Liver<br>- Beef<br>- Chicken<br>- Pork<br>- Oysters<br>- Mussels<br>- Shrimp<br>- Fish | - Pumpkin seeds<br>- Tofu<br>- Spinach<br>- Lentils<br>- Enriched cereals<br>- Eggs<br>- Quinoa<br>- Chickpeas<br>- Soybeans<br>- Nuts<br>- Blackstrap molasses |

### 2. Taking iron supplements

There are many options for taking iron supplements. They come in tablets, capsules or liquid form. Each option has different amounts of iron. Sometimes these supplements cause side effects. You may have a metallic taste in your mouth, nausea, pain in your stomach, upset stomach, vomiting, diarrhea or constipation.

To reduce the chance of side effects and get the most from your treatment, please take your iron as recommended.

**How to take them**

Pay attention to how and when you take your iron supplement. Iron needs to be taken with acidic foods or drinks to be absorbed well.

Take iron at night with an acidic drink such as:

1. Orange juice
2. Water with a lemon or a vitamin C tablet

Do not take your iron within two hours of:

1. Milk
2. Tea or Coffee (black)
3. Calcium pills
4. Pre natal vitamins
5. Any medicines

**What can help with constipation?**

Constipation is common when you are taking iron supplements as well as when you are pregnant. There are many ways to prevent constipation. Start by increasing dietary fibre and drinking lots of fluids.

If this is not enough, you can consider these medicines. They can improve your symptoms and they are considered safe in pregnancy:

1. Docusate Sodium (trade name : Colace)
2. Polyethylene glycol (PEG) 3350 (trade name : Lax-A-Day, PEG 3350, Pegalax, Relaxa)

## 3. Intra-venous (IV) iron

Sometimes, iron supplementation by mouth is not enough to raise your iron levels. In these cases, your doctor may discuss giving you iron through an IV.

## For more information

If you have any questions or concerns, please talk to your doctor or primary health care provider.
