# Supplementary material for: Development and implementation of a quality improvement toolkit, iron deficiency in pregnancy with maternal iron optimization (IRON MOM): A before-and-after study
Source: PLoS Med. 2019 Aug 20;16(8):e1002867. doi: 10.1371/journal.pmed.1002867 (PMC6701755; doi:10.1371/journal.pmed.1002867)
Supplement: S7 Fig — (PDF) [file pmed.1002867.s007.pdf]

IRONMOM

Oral Iron Formulations

- **Iron Salts* $**
  - Ferrous gluconate
    - Ferrogluc (~ $0.10/tablet)
    - Apo-Ferrous Gluconate (~ $0.10/tablet)
  - Ferrous sulfate
    - Ferodan liquid formulation (~$0.30/tsp)
    - Apo-Ferrous Sulfate (~ $0.20/tablet)
  - Ferrous fumarate
    - Eurofer (~ $0.28/capsule)
    - Palafer (~ $0.28/capsule)

- **Polysaccharide iron $$**
  - Triferex (~ $0.63/capsule)
  - Feramax (~ $0.66/capsule)
  - Polyride (~ $0.93/capsule)

- **Heme iron polypeptide**$$$** (requires multiple tablets/day)
  - Optifer Alpha (~ $0.74/tablet)
  - Proferrin (~ $1.07/tablet)

- **Intravenous iron* $$$$** (requires 2-3 infusions)
  - Iron sucrose
    - Venofer® (~ $150/infusion)

*may qualify for coverage through ODB or private insurance

**derived from bovine source
